# Comparative Analyses of Phase Noise in 28 nm CMOS LC Oscillator Circuit Topologies: Hartley, Colpitts, and Common-Source Cross-Coupled Differential Pair

**DOI:** 10.1155/2014/421321

**Published:** 2014-02-06

**Authors:** Ilias Chlis, Domenico Pepe, Domenico Zito

**Affiliations:** ^1^Tyndall National Institute, “Lee Maltings”, Dyke Parade, Cork, Ireland; ^2^Department of Electrical and Electronic Engineering, University College Cork, Cork, Ireland

## Abstract

This paper reports comparative analyses of phase noise in Hartley, Colpitts, and common-source cross-coupled differential pair LC oscillator topologies in 28 nm CMOS technology. The impulse sensitivity function is used to carry out both qualitative and quantitative analyses of the phase noise exhibited by each circuit component in each circuit topology with oscillation frequency ranging from 1 to 100 GHz. The comparative analyses show the existence of four distinct frequency regions in which the three oscillator topologies rank unevenly in terms of best phase noise performance, due to the combined effects of device noise and circuit node sensitivity.

## 1. Introduction

Oscillator phase noise (PN) is one of the main bottlenecks for the information capacity of communication systems, leading to severe challenges in the design of local oscillators in silicon technologies, especially at very high frequency [1–5]. In particular, the main difficulties are to achieve a high quality factor LC tank [6–11] and consume reasonable power [12, 13].

Oscillator phase noise has been studied extensively over the last decades [14–17]. Most of these studies are based on linear time-invariant (LTI) oscillator models, which provide important qualitative design insights, but are limited in the quantitative prediction of the power spectral density levels [18], in some cases addressed by adopting nonlinear approaches [19].

The linear time-variant (LTV) oscillator model allows a quantitative understanding of oscillator phase noise through the impulse sensitivity function (ISF), represented as Γ(*x*) [18]. Since the oscillator is assumed as a linear time-varying circuit, the phase sensitivity to noise perturbations can be described in terms of its (time-varying) impulse response.

The evaluation of the ISF involves a significant amount of transient simulations and data extractions, resulting in time consuming calculations, potentially prone to inaccuracy. Recently, new efficient frequency-domain methods operating directly in the steady-state were proposed [20, 21], allowing a consistent reduction of the simulation workload. Regardless of the methods, the analysis of the phase sensitivity can contribute significantly to a better understanding of the impact of noise sources to the oscillator phase noise in the most widespread circuit topologies.

A comparative analysis of common-source cross-coupled differential pair and differential Colpitts LC oscillators in 0.35 *μ*m CMOS technology at 2.9 GHz was carried out in [22], showing the superior performance of the cross-coupled differential topology. In this perspective, it could be interesting to extend the comparison also to other topologies, technology nodes, and oscillation frequencies. In this regard, in [23], we reported the results of a comparative analysis on Hartley, Colpitts, and common-source cross-coupled differential pair circuit topologies in 28 nm CMOS technology operating at 10 GHz, confirming the results in [22] and showing that the Colpitts topology provides superior phase noise performance with respect to the Hartley topology.

This paper reports an expansion and an extension of our preliminary comparative study of PN for the three oscillator topologies: Hartley, Colpitts, and common-source cross-coupled differential pair circuit topologies in 28 nm CMOS technology. In particular, we recap the main results and report additional aspects of the preliminary investigations; then we move forward to wider evaluations on PN contributions in relation to the operating frequency. The results of the analyses show interesting aspects not addressed by the literature. In detail, all the steps for an accurate derivation of the ISF are summarized and the PN predictions for a wide set of amplitudes of the injected current pulse are compared with the results obtained by the direct plots obtained by means of SpectreRF-Cadence Periodic Steady State (PSS) analysis. The contributions from each noise source to the overall PN are evaluated qualitatively and quantitatively through the ISF for each topology operating in a discrete set of frequencies from 1 to 100 GHz.

The paper is organized as follows.  Section 2 reports the design of the three oscillator topologies in 28 nm CMOS technology.  Section 3 summarizes the key analytical expressions for PN predictions through the ISF, the key steps, and settings for accurate evaluations and finally reports the results for the oscillation frequency of 10 GHz. In Section 4, qualitative and qualitative analyses of the PN contributed by each circuit component are carried out for each topology for a discrete set of oscillation frequencies ranging from 1 to 100 GHz.  Section 5 reports the results that reveal the existence of four different frequency regions in which the best PN performance is given case by case by a different topology. In Section 6, the conclusions are drawn.

## 2. Circuit Topologies

**Figure 1:**
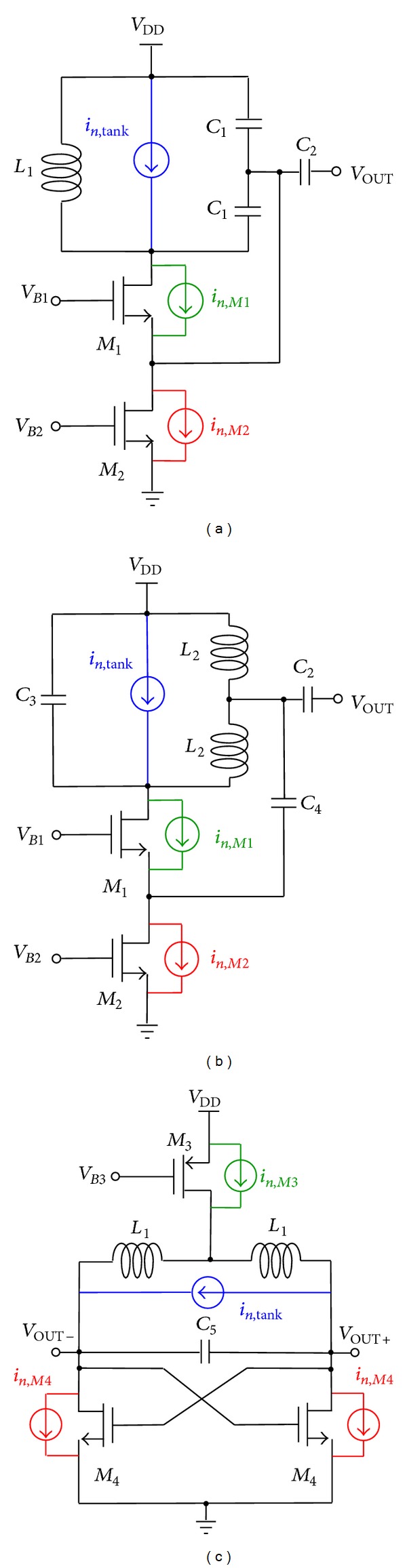
Schematic of the oscillator circuit topologies: (a) single-ended Colpitts, (b) single-ended Hartley, and (c) top-biased common-source cross-coupled differential pair. *V*_*B*1_, *V*_*B*2_, and *V*_*B*3_ are DC bias voltages. In Colpitts and Hartley topologies, the output voltage is taken after a 100 nF capacitor in order to remove the DC component.

Three LC oscillator topologies have been analysed: single-ended Colpitts, single-ended Hartley, and top-biased common-source cross-coupled differential pair oscillator topologies, as shown in Figure 1. The three oscillator circuit topologies have been implemented in 28 nm bulk CMOS technology by ST-Microelectronics by adopting the same criteria for a fair comparison as follows. The frequency of operation is 10 GHz. The sizes of the transistors and the values of the inductors and capacitors used are reported in Table 1. Despite the fact that this work is addressed to the investigations of the circuit topologies as such, rather than the circuit design and implementation, that is, regardless of the effects of parasitic components, we considered a reasonable quality factor for the LC tank in order to carry out the comparative study of the properties of each circuit topology under the same typical conditions. Thereby, a quality factor (*Q*) equal to 10 has been assumed for the inductors, considering a parasitic resistance in series with the inductor, whereas the capacitors have been considered as ideal devices. In all cases, the power consumption is 6.3 mW.

A small signal noise analysis by SpectreRF was used for the derivation of the flicker noise corner of each transistor. Assuming that the power spectral density (PSD) of the thermal and flicker noise currents generated by the transistor in the saturation region is given by (1) and (2), respectively; the flicker noise corner is given by (3) [24].

(1) $$\begin{array}{c} S_{iw}=4kT\gamma g_{m}, \end{array}$$

(2) $$\begin{array}{c} S_{if}=g_{m}^{2}\frac{k_{f}}{WLC_{\text{ox}}}\frac{1}{f^{c}}, \end{array}$$

(3) $$\begin{array}{c} f_{1/f}=\sqrt[{c}]{\frac{k_{f}}{WLC_{\text{ox}}}\frac{g_{m}}{4kT\gamma }}, \end{array}$$

where *k*_*f*_ is a bias-dependent constant, *c* is a constant with typical values ranging from 0.7 to 1.2, *C*_ox_ is the oxide capacitance per unit area, and *γ* is the excess noise coefficient. For the 28 nm bulk CMOS technology adopted, the thickness of the oxide *t*_ox_ is about 1.4 nm for the n-MOSFET and 1.7 nm for the p-MOSFET, from which we can derive that *C*_ox_ is about 0.026 and 0.02 F/m^2^, respectively. The values of *k*_*f*_, *f*_1/*f*_, and *c* have been derived by means of noise simulation of each single stand-alone transistor of Table 1. They are reported in Table 2.

## 3. Impulse Sensitivity Function

In order to get an insight of the noise contribution of each circuit component in each circuit topology, hereinafter we make use of the ISF as a predictive tool for quantitative and qualitative PN evaluations.

A detailed procedure for computation of the ISF and PN prediction in a linear time-varying system in the case of a source-coupled CMOS multivibrator with operating frequency up to 2 MHz was presented in [25]. All the results were achieved only for a single amplitude value of the injected pulse. However, the time-domain evaluation of the ISF involves a number of transient simulations, potentially prone to inaccuracy. Thereby, it is worth consolidating all the steps in order to achieve accurate results.

The impulse response from each current noise source to the oscillator output phase can be written as [18]

(4) $$\begin{array}{c} h_{\phi }\left(t,\tau \right)=\frac{\Gamma \left(\omega_{0}\tau \right)}{q_{\max}}u\left(t-\tau \right), \end{array}$$

where *q*_max⁡_ is the charge injected into a specific circuit node of the oscillator at time *t* = *τ*, *u*(*t*) is the unity step function, and Γ(*ω*_0_ *τ*) is a dimensionless periodic function that can be expressed as a Fourier series [18]:

(5) $$\begin{array}{c} \Gamma \left(\omega_{0}\tau \right)=\frac{c_{0}}{2}+\sum_{n=1}^{\infty }c_{n}\cos\left(n\omega_{0}\tau +\theta_{n}\right). \end{array}$$

The DC and root mean square (rms) values of Γ(*ω*_0_ *τ*) are given by the following two equations [18]:

(6) $$\begin{array}{c} \Gamma_{\text{DC}}=\frac{c_{0}}{2},\\ \Gamma_{\text{rms}}=\sqrt{\frac{1}{2}\sum_{n=0}^{\infty }c_{n}^{2}}. \end{array}$$

The PN of any oscillator is traditionally indicated with *L*. The thermal noise contribution to the PN spectrum, from each given noise source with a white power spectral density, can be expressed as [18]

(7) $$\begin{array}{c} L{\left\{\Delta \omega \right\}|}_{dB}=10\log\left[\frac{\Gamma_{\text{rms}}^{2}}{q_{\max}^{2}}\frac{\left(\overset{-}{i_{n}^{2}}/\Delta f\right)}{2\Delta \omega^{2}}\right], \end{array}$$

where *q*_max⁡_ is the charge injected into a circuit node by the noise source *i*_*n*_ insisting in that node and Δ*ω* is the offset from the oscillation angular frequency. Therein [18], it is tacitly assumed that *c* in (2) is equal to 1, regardless of the technology node. This assumption leads to the relatively rough but simple equation (7).

The flicker noise contribution to the PN spectrum for any oscillator, from each given noise source with a 1/*f* spectrum, can be expressed as follows [18], where *ω*_1/*f*_ is the flicker noise corner of the device:

(8) $$\begin{array}{c} L{\left\{\Delta \omega \right\}|}_{dB}=10\log\left[\frac{c_{0}^{2}}{q_{\max}^{2}}\frac{\left(\overset{-}{i_{n}^{2}}/\Delta f\right)}{8\Delta \omega^{2}}\frac{\omega_{1/f}}{\Delta \omega }\right]. \end{array}$$

### 3.1. Simulation Steps and Settings

**Figure 2:**
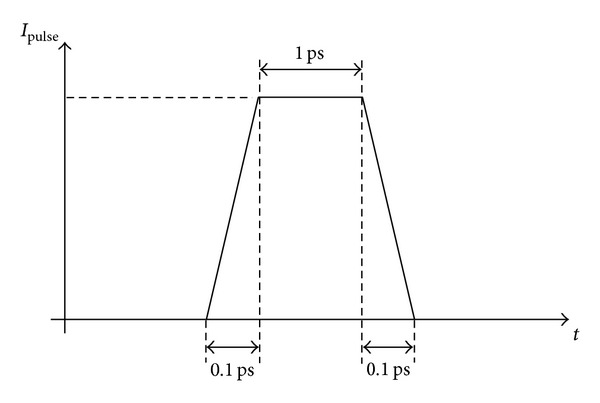
The injected current pulse.

All the simulations have been carried out by using the SpectreRF simulator in the Cadence design environment. The ISF of the oscillator topologies has been evaluated for an oscillation frequency of 10 GHz, which will be considered hereinafter as a reference for all the other cases. First we run a transient simulation in order to observe and record when the amplitude of the oscillation waveform reaches the steady state regime. In our case, this occurs with large margins after 5 ns. Afterwards, we perform other transient simulations applying current impulsive sources acting in parallel with the actual inherent current noise sources of the LC tank and transistors, by activating only one noise source at one time. The current impulses are set to occur in the steady state regime starting from a given time reference for the unperturbed solution. The pulse width of each current source has been chosen equal to 1 ps (i.e., one hundredth of the oscillation period) with 0.1 ps rise and fall time, as shown in Figure 2.

**Figure 3:**
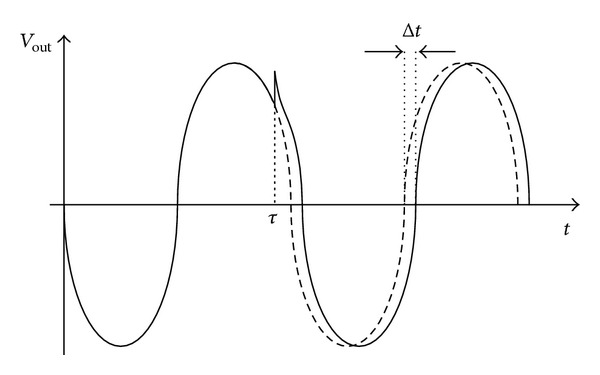
Time shift Δ*t* caused by the impulse injection occurring at the time *τ*.

The simulation has been repeated for amplitudes of the injected current of 1, 10, and 100 *μ*A and 1 and 10 mA. Each transient analysis is performed using the conservative mode and a maximum time step of 10 fs (i.e., one 10-thousandth of the oscillation period), in order to have good accuracy even in the case of the smallest injected current pulse (i.e., 1 *μ*A). The charge *q*_max⁡_ injected in each node corresponds to the area under each pulse, that is, the area of the trapezoid of Figure 2:

(9) $$\begin{array}{c} q_{\max}=I_{\text{Pulse}}\times 1.1\times 10^{-12}\;\text{Coulombs}, \end{array}$$

where *I*_pulse_ is the amplitude value of each source pulse. This is repeated for all the *N* noise sources connected in parallel and for all the *M* instants of time over one period of oscillation, where *N* = 3 and *M* = 40, in our case. The time instants have been chosen to be equally spaced in an oscillation period. The time shift caused by the impulse injection can be extracted by comparing the perturbed and unperturbed waveforms. This means that when the oscillation has reached the steady state regime, the time shift Δ*t*_*i*_ of the zero-crossing instant of the perturbed oscillation with respect to the unperturbed one, that is, when no impulse is applied, is calculated as shown in Figure 3.

Then, these time shifts are converted into phase shifts by using the following relation:

(10) $$\begin{array}{c} \Gamma \left(x=\omega_{0}t\right)=2\pi \frac{\Delta t_{i}\left(t\right)}{T}. \end{array}$$

In order to take into account the cyclostationary nature of the active device noise sources, Γ(*x*) is multiplied with *α*(*x*), where *α*(*x*) is the absolute value of the unperturbed current flowing in the respective node in which the impulses are injected, normalized to its maximum value in the period. Then, the DC and root mean square (rms) components of the product Γ(*x*) × *α*(*x*) can be calculated as follows:

(11) $$\begin{array}{c} \Gamma_{\text{DC}}=\frac{\sum_{i=1}^{40}\left[\Gamma \left(x\right)\alpha \left(x\right)\right]}{40},\\ \Gamma_{\text{rms}}=\sqrt{\frac{\sum_{i=1}^{40}\left\{{\left[\Gamma \left(x\right)\alpha \left(x\right)\right]}^{2}\right\}}{40}}. \end{array}$$

Finally, the total PN of the oscillator is computed by adding the contributions from all the noise sources acting in the circuit, according to (7) and (8). In particular, the active devices inject noise to the terms responsible for both flicker and thermal noise contributions to the oscillator PN, whereas the LC tank participates only to the thermal noise contribution to PN. Equation (12) gives the total PN for each of the three oscillators, where *m* is the number of transistors of the oscillator circuit. The first sum in (12) describes the PN component contributed by the thermal noise. As a result, it contains an additional term (*m* + 1), due to thermal noise coming from the LC tank:

(12) L{Δω}|dB=10log⁡{∑i=1m+1[Γrms2qmax⁡2(in2−/Δf)i2Δω2]+∑i=1m[ΓDC2qmax⁡2(in2−/Δf)i8Δω2(ω1/f)iΔω]}.

### 3.2. Results

**Figure 4:**
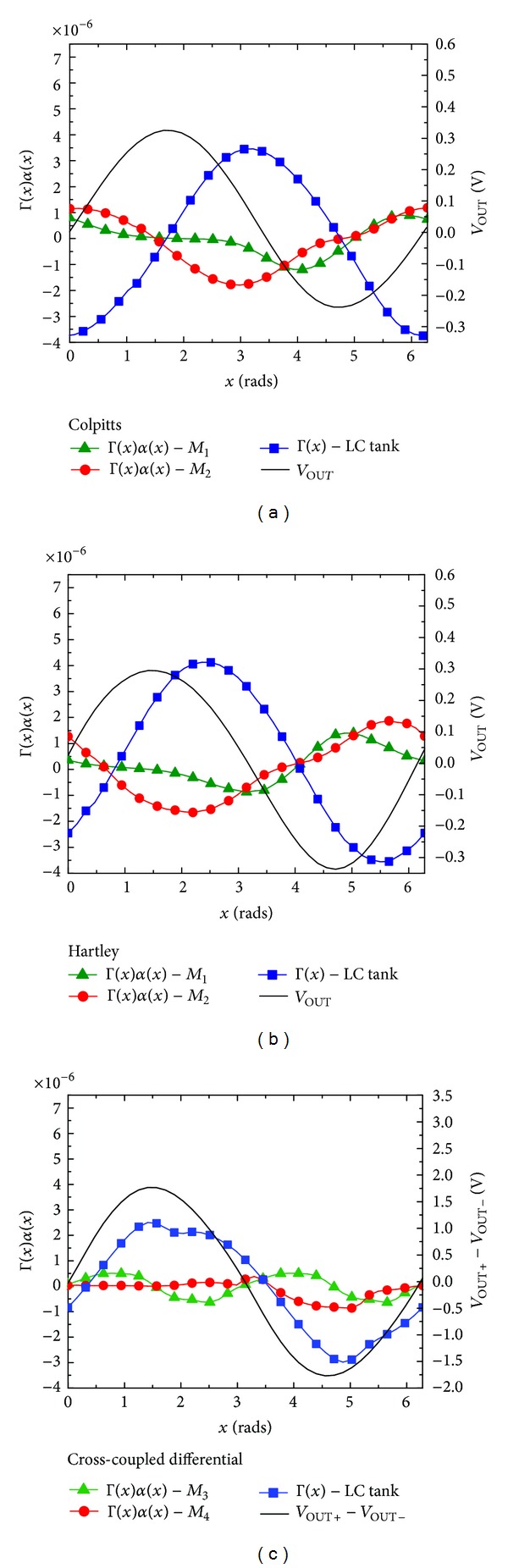
Γ(*x*)*α*(*x*) of the MOSFETs and Γ(*x*) of the LC tank versus phase for a 1 *μ*A amplitude current impulse, for the oscillation frequency of 10 GHz: (a) Colpitts topology, (b) Hartley topology, and (c) common-source cross-coupled differential pair topology.

Figure 4 reports Γ(*x*)*α*(*x*) for an injected current pulse amplitude of 1 *μ*A versus the phase for the injected noise sources, during one oscillation period, for the three oscillator circuit topologies.

**Figure 5:**
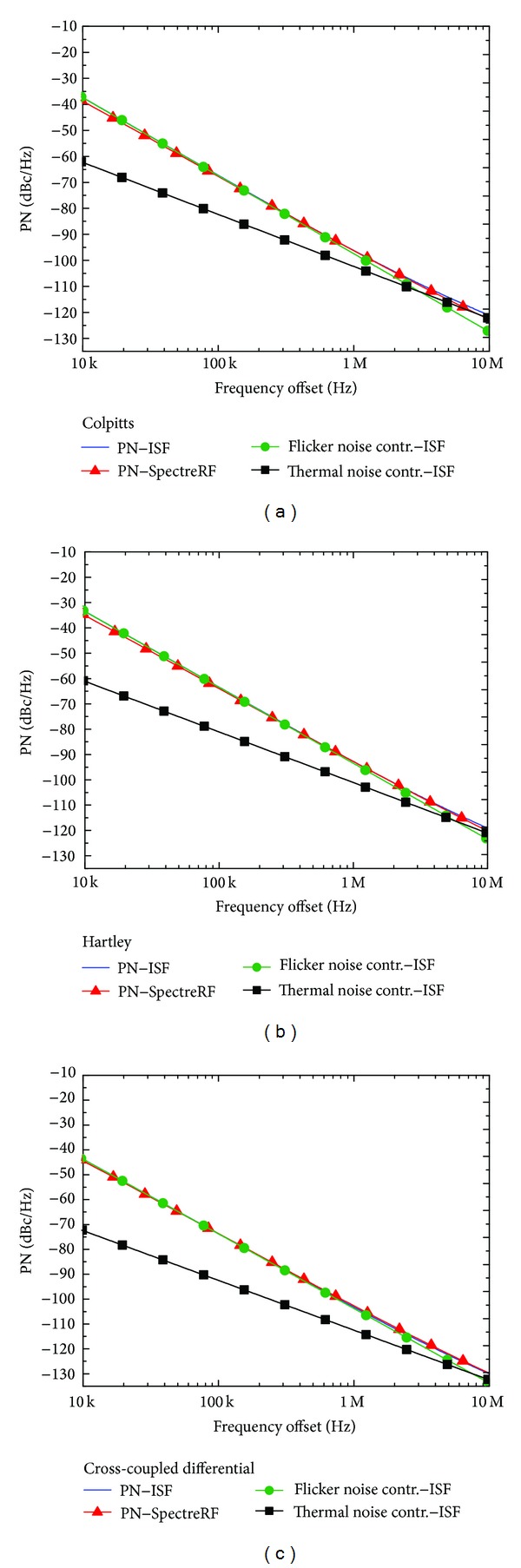
PN versus frequency offset for the three oscillator circuit topologies, obtained through the ISF for a 1 *μ*A current impulse and direct plot from PSS and periodic noise (Pnoise) SpectreRF simulations, for the oscillation frequency of 10 GHz. The flicker and thermal noise contributions to the overall PN are also plotted in order to identify the 1/*f* ^3^ PN frequency corner. (a) Colpitts: the 1/*f* ^3^ PN corner is at the frequency offset of 3.1 MHz. (b) Hartley: the 1/*f* ^3^ PN corner is at the frequency offset of 5.7 MHz. (c) Common-source cross-coupled differential pair: the 1/*f* ^3^ PN corner is at the frequency offset of 7.5 MHz.

Figure 5 reports the comparison between the PN obtained through the ISF and the PN obtained by direct plots from PSS and periodic noise simulations, for the three oscillator circuit topologies. Note that the PN predicted by the ISF is very close to the values obtained by means of SpectreRF simulations.  Table 3 provides the PN results for all the current impulse amplitude values, for a 1 MHz frequency offset from the carrier.

Note that for this oscillation frequency (10 GHz) the PN of the common-source cross-coupled differential pair topology is lower to the PN of the Colpitts topology, in agreement with [22], and that the PN of Colpitts is lower to the PN exhibited by the Hartley topology. Moreover, note that the agreement degrades for higher pulse amplitudes, when the current-to-phase transfer function starts becoming nonlinear. The amplitude in which this occurs is slightly different for each oscillator topology, but for the injected current impulse of 1 *μ*A, the difference between the PN predicted by the ISF method and the one given by PSS and periodic noise (Pnoise) analysis is lower than 1% at a 1 MHz frequency offset.

## 4. Analyses and Comparison versus Oscillation Frequency

The investigations through the ISF can provide a better understanding of the PN in each oscillator topology. In order to be able to extract further useful considerations about the devices and topologies, the previous analyses have been reiterated also for other oscillation frequencies. In detail, the three oscillator topologies have been implemented also for 1 and 100 GHz operations, by keeping the quality factor of 10 for the LC tank and preserving the same power consumption of 6.3 mW as in the case of the 10 GHz oscillation frequency. The transistor sizes were also kept the same as in the previous case. As a consequence of the results reported in the previous section, we injected noise current impulses with amplitude of 1 *μ*A.

Table 4 reports the values of the individual circuit components for the topologies of Figure 1, used for the oscillation frequencies of 1 and 100 GHz.

Table 5 reports the PN values at a 1 MHz offset predicted by the ISF along with the values obtained by means of SpectreRF simulations for the oscillation frequencies of 1 and 100 GHz.

**Figure 6:**
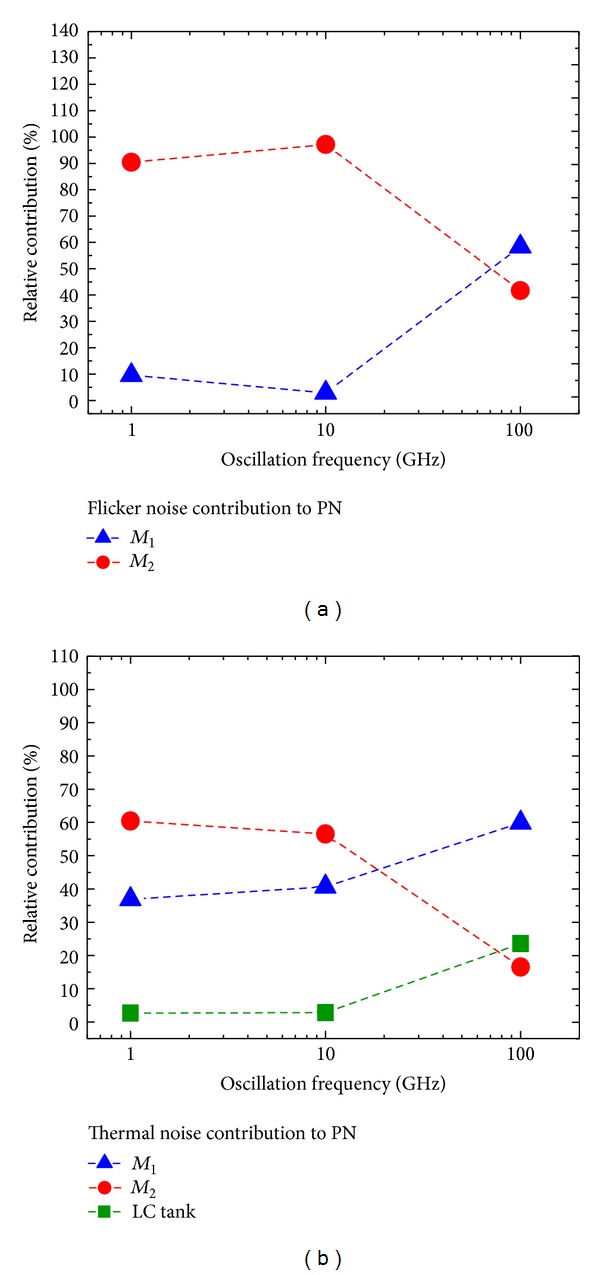
Relative contributions of *M*_1_, *M*_2_, and the LC tank for the Colpitts topology versus oscillation frequency @ 1 MHz offset. (a) Flicker noise contribution to PN. (b) Thermal noise contribution to PN.

Figure 6 reports the relative contributions of *M*_1_, *M*_2_, and LC tank to the overall PN versus the oscillation frequency for the Colpitts topology, in both the flicker and thermal noise contributions to PN.

**Figure 7:**
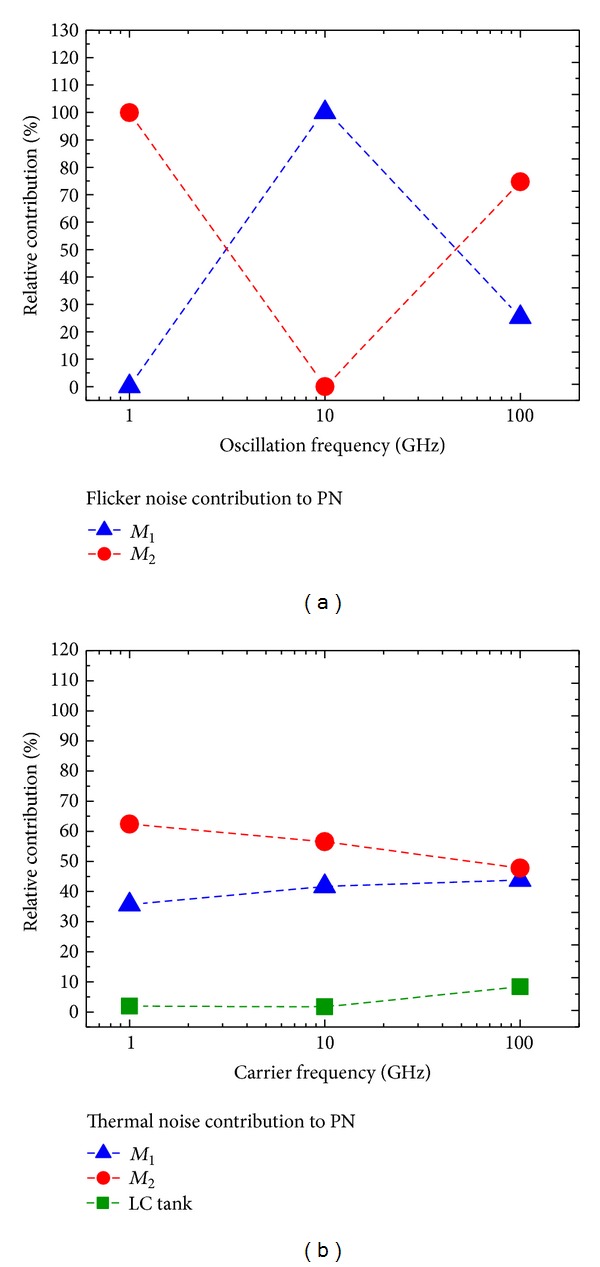
Relative contributions of *M*_1_, *M*_2_, and the LC tank for the Hartley topology versus oscillation frequency @ 1 MHz offset. (a) Flicker noise contribution to PN. (b) Thermal noise contribution to PN.

**Figure 8:**
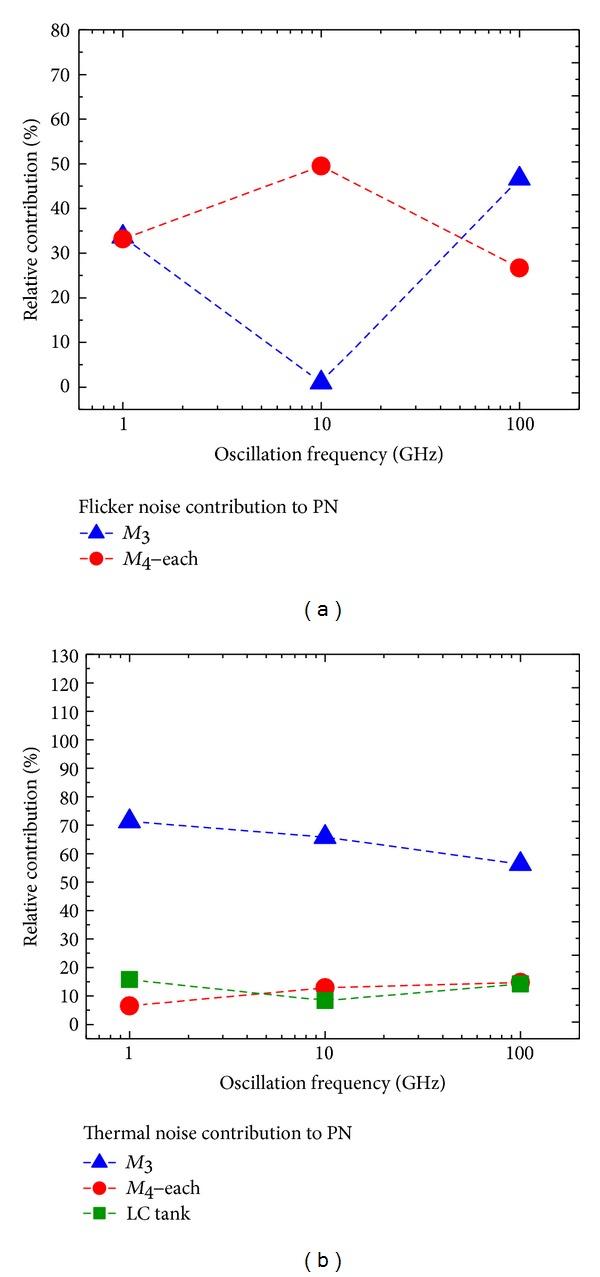
Relative contributions of *M*_3_, *M*_4_, and the LC tank for the common-source cross-coupled differential pair topology versus frequency of oscillation @ 1 MHz offset. (a) Flicker noise contribution to PN. (b) Thermal noise contribution to PN.

Figures 7 and 8 report the results for the Hartley and common-source cross-coupled differential pair topologies, respectively.

### 4.1. Comparative Analysis between Devices

The relative contributions to the overall current flicker and thermal noise from MOSFETs and LC tank of the three oscillator topologies are summarized in Table 6, as well as the values of Γ_DC_ and Γ_rms_ calculated for the 1 *μ*A injected noise source.

These results stimulate some careful evaluations about the noise contributions of each device in each oscillator topology at different oscillation frequencies.

To do this, we could refer again to (7), (8), and (12) and consider preliminarily that the amount of flicker or thermal noise of the transistor in a certain region of operation does not determine exclusively the flicker or thermal noise contribution to the oscillator PN, as reported in [18]. In particular, we can observe that, for a given *q*_max⁡_ (9) and a given frequency offset Δ*ω* = 2*π* × 10^6^, the amount of flicker noise contribution to PN is proportional to the product of the transistor flicker noise and Γ_DC_^2^:

(13) $$\begin{array}{c} L\left\{\Delta \omega \right\}\propto \sum_{i=1}^{m}\left\{\Gamma_{\text{DC}}^{2}\left[\left(\frac{\overset{-}{i_{n}^{2}}}{\Delta f}\right)\frac{\omega_{1/f}}{\Delta \omega }\right]\right\}, \end{array}$$

whereas the amount of thermal noise contribution to PN is proportional to the product of the thermal noise of the transistor and LC tank and their respective Γ_rms_^2^:

(14) $$\begin{array}{c} L\left\{\Delta \omega \right\}\propto \sum_{i=1}^{m+1}\left\{\Gamma_{\text{rms}}^{2}\left(\frac{\overset{-}{i_{n}^{2}}}{\Delta f}\right)\right\}. \end{array}$$

In other words, the flicker noise is weighted by Γ_DC_^2^, whereas the thermal noise is weighted by Γ_rms_^2^, as mentioned in [18]. On the other hand, Γ_DC_ and Γ_rms_ do not depend on the device noise sources but on the node in which the noise current is injected in a circuit topology.

Considering these aspects, it is worth highlighting the following observations on the above results.

In the Colpitts topology, we observe from Figure 6(a) and Table 6 that, for oscillation frequencies higher than about 70 GHz, transistor *M*_1_ dominates the flicker noise contribution to PN. However, *M*_2_ dominates at frequencies lower than 70 GHz, despite the fact that *M*_2_ generates a lower flicker noise than *M*_1_. This is due to the fact that, according to Table 6, the absolute value of Γ_DC_ for *M*_2_ is larger than Γ_DC_ for *M*_1_ at low frequencies of oscillation. In other terms, this means that the oscillation waveform at the node (drain node of *M*_2_) into which the noise current is injected is less symmetrical with respect to the rise and fall times [18]. Regarding the thermal noise contribution to PN, shown in Figure 6(b), at oscillation frequencies above 20 GHz, *M*_1_ has the major PN contribution. However, below 20 GHz, *M*_2_ has a higher Γ_rms_ than *M*_1_, as shown in Table 6. As a result, it takes a larger portion of the thermal noise contribution to PN at oscillation frequencies below 20 GHz. This happens despite the thermal noise contribution of *M*_2_ is half that of *M*_1_.

As for the Hartley oscillator topology, we observe from Figure 7(a) that, at oscillation frequencies between 3 and 50 GHz, transistor *M*_1_ dominates the flicker noise contribution to PN. Nonetheless, in lower and higher oscillation frequencies, *M*_2_ dominates, despite its lower flicker noise with respect to *M*_1_ as shown in Table 6, since its contribution is characterized by a higher absolute value of Γ_DC_, as again shown in Table 6. In the thermal noise contribution to PN reported in Figure 7(b), *M*_2_ presents the major contribution, because, from Table 6, *M*_2_ has a higher Γ_rms_ than *M*_1_. Thereby, it takes a larger portion of the thermal noise contribution to PN. This happens regardless of the fact that the thermal noise contribution of *M*_2_ is half that of *M*_1_, according to Table 6.

As for the common-source cross-coupled differential pair oscillator topology, we see in Figure 8(a) that the pair of n-MOSFETs *M*_4_, at frequencies lower than 50 GHz, is responsible for most of the flicker noise contributions to PN, as it not only generates more flicker noise but also has a higher absolute value of Γ_DC_ than *M*_3_ (see Table 6). After 50 GHz, the contribution of *M*_3_ increases due to its higher Γ_DC_ value and surpasses that of *M*_4_, even though *M*_4_ generates a higher flicker noise. With respect to the behavior of the thermal noise contribution to PN seen in Figure 8(b), the relative contribution from the current source *M*_3_ gradually drops with increasing oscillation frequencies, whereas *M*_4_ follows an opposite trend.

Moreover, from Figures 6(b) and 7(b), we note that, in the Colpitts and Hartley topologies, the LC tank occupies at lower oscillation frequencies a small portion of the contribution of thermal noise to PN graph, because, as Table 6 indicates, the thermal noise generated by the LC tank is at least one order of magnitude below the thermal noise generated by the transistors in each case. However, both in Colpitts and Hartley, the contribution of the LC tank increases at higher oscillation frequencies, where Table 6 indicates that Γ_rms_ of the tank is notably larger than Γ_rms_ of both devices.

On the other hand, from Figures 6(b), 7(b), and 8(b), we note that, in all three oscillator topologies, the relative contribution of the current sources *M*_2_ and *M*_3_ to the thermal noise contribution to PN drops at higher oscillation frequencies. According to Table 6, this is due to the reduction of the Γ_rms_ for the current sources relative to the Γ_rms_ values for the other oscillator components.

### 4.2. Comparative Analysis between Topologies

By using the values in Table 6 along with (13) and (14), we can determine the flicker and thermal noise contributions obtained by the ISF for a 1 *μ*A injected current source, as reported in Table 7.

**Figure 9:**
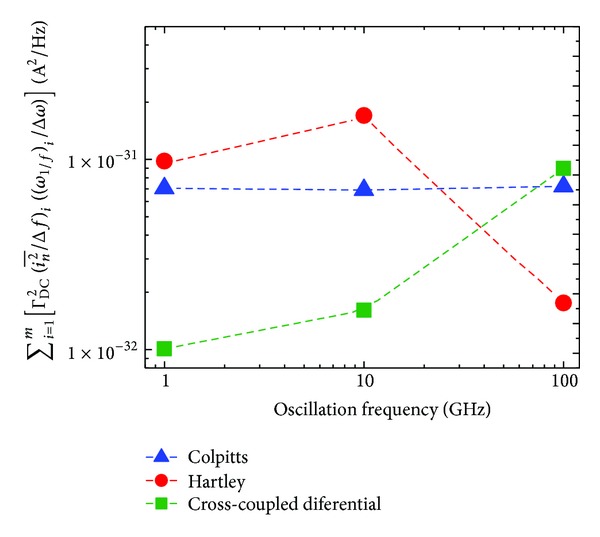
Sum of $\Gamma_{\text{DC}}^{2}\left[\left(\overset{-}{i_{n}^{2}}/\Delta f\right)\left(\omega_{1/f}/\Delta \omega \right)\right]$ for all flicker noise sources in each oscillator topology @ 1 MHz offset versus oscillation frequency for Colpitts, Hartley, and common-source cross-coupled differential pair topologies.

**Figure 10:**
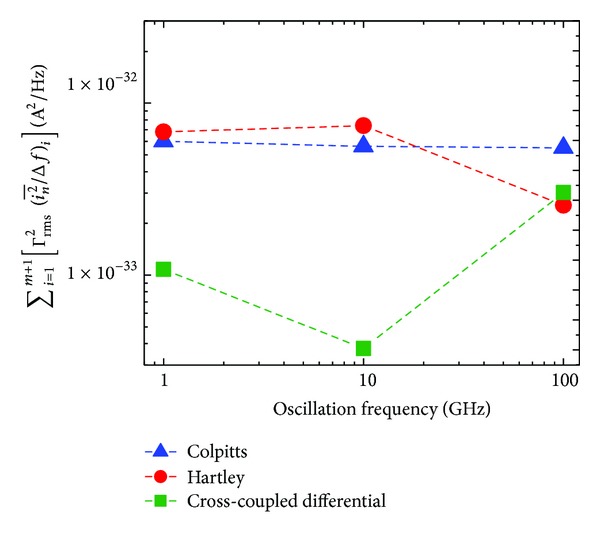
Sum of $\Gamma_{\text{rms}}^{2}\left(\overset{-}{i_{n}^{2}}/\Delta f\right)$ for all thermal noise sources in each oscillator topology @ 1 MHz offset versus oscillation frequency for Colpitts, Hartley, and common-source cross-coupled differential pair topologies.

In order to provide them in a more intuitive form, the results in Table 7 are plotted in Figures 9 and 10.

As in the previous section, by considering a given *q*_max⁡_ (9) and a given frequency offset Δ*ω* = 2*π* × 10^6^, (13) and (14) can be used in order to compare the flicker and thermal noise contributions, respectively, to the PN spectrum of various oscillator topologies. In this perspective, Figures 9 and 10 show the variation of the flicker and thermal noise contributions to PN, respectively, for the three oscillator topologies under investigation with respect to changes in the oscillation frequency, at a frequency offset of 1 MHz.

## 5. Topology Performances versus Oscillation Frequency Regions

In the previous section, we reported the results of the effective ISF for every active device of the three oscillator topologies according to (11). Here we try to explain the different PN behavior achieved for the three oscillator topologies over the frequency range from 1 to 100 GHz. The results of the previous section suggest considering additional oscillation frequencies. For this reason, the three topologies have been designed also for the additional oscillation frequencies of 30, 50, and 70 GHz, according to the same criteria of Sections 2 and 4.

Table 8 reports the values of the circuit components for each topology for the oscillation frequencies of 30, 50, and 70 GHz.

**Figure 11:**
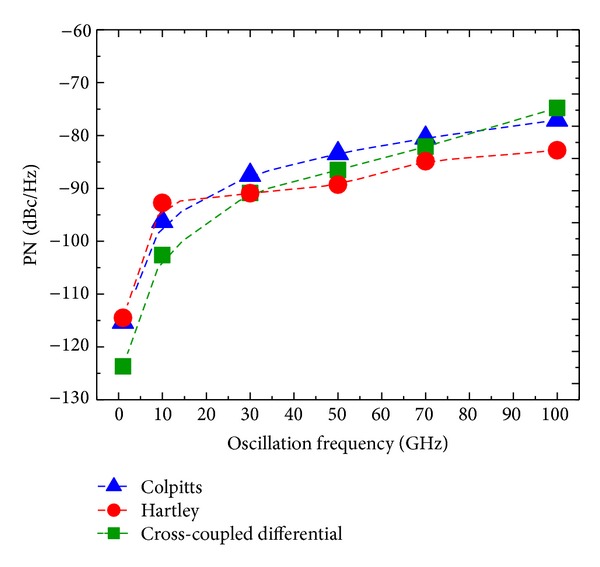
PN at a 1 MHz frequency offset from carrier versus oscillation frequency for Colpitts, Hartley, and common-source cross-coupled differential pair topologies by SpectreRF.

Figure 11 reports the PN results obtained by SpectreRF for 1, 10, 30, 50, 70, and 100 GHz at a 1 MHz frequency offset from the carrier.

These results allow us to identify the following four main frequency regions: 1–20, 20–30, 30–80, and 80–100 GHz. They offer the opportunity to carry out further comparative analyses and derive a number of observations.

Comparing the total (i.e., sum) contributions of the flicker and thermal noise sources in Table 7 and Figures 9 and 10, we can note that the flicker noise contribution dominates at the frequency offset of 1 MHz at the oscillation frequencies of 1, 10, and 100 GHz. We can also note that term (13) determining the flicker noise contribution derived from ISF, as in Figure 9, shows an agreement with the PN derived by SpectreRF-Cadence, as in Figure 11.

This is because, as already mentioned in Section 4, from (7), (8), and (12), it can be concluded that the flicker noise contribution to PN is defined by the product of the transistor flicker noise with Γ_DC_^2^, as expressed in (13) and quantified in Table 7.

*Region 1 (1–20 GHz)*. According to Figure 11, the common-source cross-coupled differential pair topology exhibits the lowest PN with respect to the other two topologies. The highest PN is exhibited by the Hartley topology. This is in agreement with the trend reported in Figure 9. Delving into the separate noise sources as addressed in Section 4 and shown in Figures 6(a), 7(a), and 8(a), the nodes mostly prone to the current noise injection are the drain of *M*_2_ in the Colpitts topology, the drain of *M*_2_ from 1 to 3 GHz and the drain of *M*_1_ from 3 to 20 GHz in the Hartley topology, and the drain of both *M*_3_ and *M*_4_ in the common-source cross-coupled differential pair topology.

*Region 2 (20–30 GHz)*. In this region, we note from Figure 11 that the common-source cross-coupled differential pair topology still maintains the best PN performance, but, unlike the above case, we can observe an inversion between the Hartley and Colpitts topologies. The latter exhibits the worst PN at 30 GHz. Figure 9 follows approximately the same results. From Figures 6(a), 7(a), and 8(a), we can see that the nodes mostly sensitive to noise injections are the drain of *M*_2_ in the Colpitts topology, the drain of *M*_1_ in the Hartley topology, and the drain of *M*_4_ in the common-source cross-coupled differential pair topology.

*Region 3 (30–80 GHz)*. In Figure 11, we register an inversion for the best PN performance, given now by the Hartley topology, whereas the Colpitts topology still exhibits the worst PN as in the previous case. A similar behavior is exhibited in Figure 9. In this region the nodes mostly sensitive to noise injection according to Figures 6(a), 7(a), and 8(a) are: the drain of *M*_2_ in the Colpitts topology, the drain of *M*_1_ up to 50 GHz and the drain of *M*_2_ at higher frequencies in the Hartley topology, and the drain of both *M*_3_ and *M*_4_ until 50 GHz and of *M*_3_ above 50 GHz in the common-source cross-coupled differential pair topology.

*Region 4 (80–100 GHz)*. Figure 11 indicates that Hartley continues to exhibit the lowest PN. However, with respect to the previous case, here we can observe an inversion of performance between the Colpitts topology and the common-source cross-coupled differential pair topology, which now exhibits the highest PN. We can also derive the same conclusions from Figure 9. The operation in the triode region for some parts of the oscillation period is the main reason for this noise performance degradation at the highest frequencies in the common-source cross-coupled differential pair topology according to the notes in [26]. Indeed, our design operates in the voltage-limited regime, thus causing the active devices to enter in the triode region at the peaks of the differential output node voltage. We notice from Figures 6(a), 7(a), and 8(a) that the most sensitive nodes in this frequency range are the drain of *M*_1_ in the Colpitts topology, the drain of *M*_2_ in the Hartley topology; and the drain of *M*_3_ in the common-source cross-coupled differential pair topology.

At least up to a 1 MHz frequency offset from the carrier, the flicker noise contribution is dominant according to Figures 5(a)–5(c) and Table 7. Therefore, the proportional increase of the flicker noise contribution to PN due to *M*_3_ at the highest oscillation frequencies in the common-source cross-coupled differential pair topology, as observed in Table 6 and Figure 8(a), is the main cause of the overall PN increase. Actually, this is an effect of the losses through the p-MOSFET tail current source that become part of the tank circuit, thus impairing its *Q* [27, 28]. Note that the superior PN performance of the Hartley topology at high frequencies noted in Regions 3 and 4 is in agreement with the observations in [26, 29].

## 6. Conclusions

PN comparative analyses have been carried out for Colpitts, Hartley, and common-source cross-coupled differential pair LC oscillator topologies in the frequency range from 1 to 100 GHz. The circuit topologies have been implemented in 28 nm bulk CMOS technology for operation at 1, 10, 30, 50, 70, and 100 GHz, maintaining equal power consumption, quality factor, and transistor sizes for a fair comparison among all the circuit topologies. All the steps and settings for accurate evaluations of the impulse sensitivity function have been discussed and clarified in depth. PN performances have also been evaluated directly through periodic steady-state simulations in the SpectreRF-Cadence environment. These last results have been compared with the results obtained through the ISF for a wide set of amplitudes of injected current pulses. The PN predicted by the ISF is in good agreement with the results obtained by SpectreRF under the given simulation settings, especially for the pulse amplitude of 1 *μ*A.

Moreover, the investigations on the PN contributions from each component of the investigated oscillator circuit topologies have been reported and discussed in detail. The results show that, under the adopted design conditions, the three oscillator topologies rank unevenly in terms of the best PN performance rating scale for oscillation frequencies from 1 to 100 GHz. This comes as a result of the frequency dependence of both contributions from each circuit component and the sensitivity to noise injections in the circuit nodes. Recent studies refer to the common-source cross-coupled differential pair topology as the one with the best PN as a consequence of the circuit designs carried out at lower frequencies. Our comparative analyses reported here show that there is no superior topology in the absolute sense, but that the identification of the best circuit topology with respect to PN is strictly related to the operating frequency range. Nowadays, the most popular topology used is the common-source cross-coupled differential mainly due to its reliable startup. However, the results presented here, suggest the opportunity to invest additional studies and efforts in exploring the circuit design implementations also of other topologies. The potential of the latter may have been perhaps underestimated until today, especially at very high frequencies. Nowadays, thanks to the recent advances in the nano-scale technology process, MOSFETs with cut-off and max frequencies in excess of 280 and 350 GHz [30], respectively, are available. Their potential use involves a number of emerging wireless applications in the millimeter-wave frequency range.

## Figures and Tables

**Table 1:** Device sizing.

Transistor width (*μ*m)				Capacitor value (pF)					Inductor value (pH)	
*M*_1_	*M*_2_	*M*_3_	*M*_4_	*C*_1_	*C*_2_	*C*_3_	*C*_4_	*C*_5_	*L*_1_	*L*_2_
30	30	30	15	0.97	10^5^	0.495	0.8	0.229	500	250

**Table 2:** Flicker noise modeling.

Transistor	*k*_*f*_ (V^2^ F)	*f*_1/*f*_ (MHz)	*c *
*M*_1_	1.09 × 10^−22^	1390	0.9
*M*_2_	2.26 × 10^−23^	870	0.9
*M*_3_	1.88 × 10^−23^	1100	0.93
*M*_4_	1.25 × 10^−22^	1430	0.9

**Table 3:** Summary of the PN results obtained by SpectreRF and ISF.

PN (dBc/Hz) @ 1 MHz frequency offset						
Topology	SpectreRF	ISF				
		1 *μ*A	10 *μ*A	100 *μ*A	1 mA	10 mA
Colpitts	−96.25	−96.20	−98.33	−98.49	−98.50	−98.45
Hartley	−92.75	−92.79	−95.18	−94.36	−94.85	−95.29
Cross coupled	−102.66	−102.69	−102.84	−102.83	−102.84	−102.94

**Table 4:** Device sizing for oscillation frequencies of 1 and 100 GHz.

Frequency (GHz)	Transistor width (*μ*m)				Capacitor value (pF)					Inductor value (pH)	
	*M*_1_	*M*_2_	*M*_3_	*M*_4_	*C*_1_	*C*_2_	*C*_3_	*C*_4_	*C*_5_	*L*_1_	*L*_2_
1	30	30	30	15	10	10^5^	5	10	2.5	5 × 10^3^	2.5 × 10^3^
100	30	30	30	15	0.0515	10^5^	0.023	0.1	0.0057	50	25

**Table 5:** Summary of the PN results obtained by SpectreRF and ISF.

PN (dBc/Hz) @ 1 MHz frequency offset				
Topology	1 GHz		100 GHz	
	SpectreRF	ISF (1 *μ*A)	SpectreRF	ISF (1 *μ*A)
Colpitts	−115.31	−116	−77.06	−77.69
Hartley	−114.52	−114.85	−81.18	−81.38
Cross coupled	−123.7	−124.06	−74.78	−75.59

**Table 6:** Γ_DC_, Γ_rms_, and relative noise contributions for a 1 *μ*A injected noise source @ 1 MHz offset.

Colpitts	Contribution to total flicker noise (%)	Contribution to total thermal noise (%)	Γ_DC_			Γ_rms_		
Oscillation freq.			1 GHz	10 GHz	100 GHz	1 GHz	10 GHz	100 GHz

*M*_1_	76.88	67.18	3.8 × 10^−8^	−2.0 × 10^−8^	5.4 × 10^−7^	5.9 × 10^−7^	5.9 × 10^−7^	1.1 × 10^−6^
*M*_2_	23.1	32.27	−2.0 × 10^−7^	−2.0 × 10^−7^	9.9 × 10^−8^	1.1 × 10^−6^	1.0 × 10^−6^	7.9 × 10^−7^
LC tank		0.55				2.6 × 10^−6^	1.8 × 10^−6^	6.9 × 10^−6^

Hartley	Contribution to total flicker noise (%)	Contribution to total thermal noise (%)	Γ_DC_			Γ_rms_		

Oscillation freq.			1 GHz	10 GHz	100 GHz	1 GHz	10 GHz	100 GHz

*M*_1_	76.88	67.18	−4.3 × 10^−9^	1.9 × 10^−7^	1.5 × 10^−7^	6.1 × 10^−7^	7.0 × 10^−7^	5.4 × 10^−7^
*M*_2_	23.1	32.27	−3.0 × 10^−7^	−4.1 × 10^−9^	−3.0 × 10^−7^	1.1 × 10^−6^	1.2 × 10^−6^	6.4 × 10^−7^
LC tank		0.55				2.5 × 10^−6^	2.7 × 10^−6^	2.0 × 10^−6^

Cross coupled	Contribution to total flicker noise (%)	Contribution to total thermal noise (%)	Γ_DC_			Γ_rms_		

Oscillation freq.			1 GHz	10 GHz	100 GHz	1 GHz	10 GHz	100 GHz

*M*_3_	20.60	26.47	−3.3 × 10^−8^	−1.5 × 10^−8^	3.3 × 10^−7^	4.5 × 10^−7^	4.1 × 10^−7^	6.7 × 10^−7^
*M*_4_	39.7-each	30.70-each	4.6 × 10^−8^	−1.5 × 10^−7^	2.0 × 10^−7^	2.7 × 10^−7^	3.7 × 10^−7^	6.7 × 10^−7^
LC tank		12.13				1.8 × 10^−6^	1.8 × 10^−6^	2.9 × 10^−6^

**Table 7:** Noise contributions @ 1 MHz frequency offset for a 1 *μ*A injected noise current.

Colpitts	$\Gamma_{\text{DC}}^{2}\left[\left(\frac{\bar{i_{n}^{2}}}{\Delta f}\right)\frac{\omega_{1/f}}{\Delta \omega }\right]$			$\Gamma_{\text{rms}}^{2}\left(\frac{\bar{i_{n}^{2}}}{\Delta f}\right)$		
Oscillation freq.	1 GHz	10 GHz	100 GHz	1 GHz	10 GHz	100 GHz

*M*_1_	6.8 × 10^−33^	2.0 × 10^−33^	4.2 × 10^−32^	2.2 × 10^−33^	2.3 × 10^−33^	3.3 × 10^−33^
*M*_2_	6.4 × 10^−32^	6.7 × 10^−32^	3.0 × 10^−32^	3.6 × 10^−33^	3.2 × 10^−33^	9.1 × 10^−34^
LC tank	* *			1.6 × 10^−34^	1.6 × 10^−34^	1.3 × 10^−33^
Total (Σ)	7.1 × 10^−32^	6.9 × 10^−32^	7.2 × 10^−32^	6.0 × 10^−33^	5.6 × 10^−33^	5.5 × 10^−33^

Hartley	$\Gamma_{\text{DC}}^{2}\left[\left(\frac{\bar{i_{n}^{2}}}{\Delta f}\right)\frac{\omega_{1/f}}{\Delta \omega }\right]$			$\Gamma_{\text{rms}}^{2}\left(\frac{\bar{i_{n}^{2}}}{\Delta f}\right)$		

Oscillation freq.	1 GHz	10 GHz	100 GHz	1 GHz	10 GHz	100 GHz

*M*_1_	6.2 × 10^−35^	1.7 × 10^−31^	4.4 × 10^−33^	2.4 × 10^−33^	3.1 × 10^−33^	1.1 × 10^−33^
*M*_2_	9.8 × 10^−32^	2.7 × 10^−35^	1.3 × 10^−32^	4.2 × 10^−33^	4.2 × 10^−33^	1.2 × 10^−33^
LC tank	* *			1.3 × 10^−34^	1.3 × 10^−34^	2.1 × 10^−34^
Total (Σ)	9.8 × 10^−32^	1.7 × 10^−31^	1.8 × 10^−32^	6.8 × 10^−33^	7.4 × 10^−33^	2.5 × 10^−33^

Cross coupled	$\Gamma_{\text{DC}}^{2}\left[\left(\frac{\bar{i_{n}^{2}}}{\Delta f}\right)\frac{\omega_{1/f}}{\Delta \omega }\right]$			$\Gamma_{\text{rms}}^{2}\left(\frac{\bar{i_{n}^{2}}}{\Delta f}\right)$		

Oscillation freq.	1 GHz	10 GHz	100 GHz	1 GHz	10 GHz	100 GHz

*M*_3_	3.4 × 10^−33^	1.3 × 10^−34^	4.2 × 10^−32^	7.7 × 10^−34^	5.1 × 10^−34^	1.7 × 10^−33^
*M*_4_	6.7 × 10^−33^	1.3 × 10^−32^	4.8 × 10^−32^	1.4 × 10^−34^	2.0 × 10^−34^	8.9 × 10^−34^
LC tank	* *			1.7 × 10^−34^	6.5 × 10^−35^	4.3 × 10^−34^
Total (Σ)	1.0 × 10^−32^	1.3 × 10^−32^	9.0 × 10^−32^	1.1 × 10^−33^	7.8 × 10^−34^	3.0 × 10^−33^

**Table 8:** Device sizing for oscillation frequencies of 30, 50, and 70 GHz.

Frequency (GHz)	Transistor width (*μ*m)				Capacitor value (pF)					Inductor value (pH)	
	*M*_1_	*M*_2_	*M*_3_	*M*_4_	*C*_1_	*C*_2_	*C*_3_	*C*_4_	*C*_5_	*L*_1_	*L*_2_
30	30	30	30	15	0.286	10^5^	0.1385	0.4	0.0627	166.7	83.35
50	30	30	30	15	0.15	10^5^	0.071	0.25	0.0297	100	50
70	30	30	30	15	0.0928	10^5^	0.0439	0.15	0.0158	71.4	35.7
